# Hybridization in East African swarm-raiding army ants

**DOI:** 10.1186/1742-9994-8-20

**Published:** 2011-08-22

**Authors:** Daniel JC Kronauer, Marcell K Peters, Caspar Schöning, Jacobus J Boomsma

**Affiliations:** 1 Centre for Social Evolution, Department of Biology, University of Copenhagen, Universitetsparken 15, 2100 Copenhagen, Denmark; 2 Laboratory of Insect Social Evolution, The Rockefeller University, 1230 York Avenue, New York, NY 10065, USA; 3 Zoological Research Museum Alexander Koenig, Adenauerallee 160, 53113 Bonn, Germany; 4 Department of Animal Ecology and Tropical Biology, Biocenter, University of Würzburg, Am Hubland, 97074 Würzburg, Germany; 5 Länderinstitut für Bienenkunde, Friedrich-Engels-Strasse 32, 16540 Hohen Neuendorf, Germany

**Keywords:** Dorylinae, Formicidae, introgression, microsatellites, mtDNA, gene flow

## Abstract

**Background:**

Hybridization can have complex effects on evolutionary dynamics in ants because of the combination of haplodiploid sex-determination and eusociality. While hybrid non-reproductive workers have been found in a range of species, examples of gene-flow via hybrid queens and males are rare. We studied hybridization in East African army ants (*Dorylus *subgenus *Anomma*) using morphology, mitochondrial DNA sequences, and nuclear microsatellites.

**Results:**

While the mitochondrial phylogeny had a strong geographic signal, different species were not recovered as monophyletic. At our main study site at Kakamega Forest, a mitochondrial haplotype was shared between a "*Dorylus molestus*-like" and a "*Dorylus wilverthi*-like" form. This pattern is best explained by introgression following hybridization between *D. molestus *and *D. wilverthi*. Microsatellite data from workers showed that the two morphological forms correspond to two distinct genetic clusters, with a significant proportion of individuals being classified as hybrids.

**Conclusions:**

We conclude that hybridization and gene-flow between the two army ant species *D. molestus *and *D. wilverthi *has occurred, and that mating between the two forms continues to regularly produce hybrid workers. Hybridization is particularly surprising in army ants because workers have control over which males are allowed to mate with a young virgin queen inside the colony.

## Background

While botanists have long accepted that hybridization plays an important role in plant evolution and regularly leads to the emergence of new species [1], zoologists have traditionally regarded hybridization and interspecific gene flow as rare exceptions [2]. However, the advent of molecular genetic markers has changed this view during the last decade and it is now widely accepted that hybridization between closely related species is also common in animals [3-5]. Hybrids are often non-viable or sterile due to negative epistasis and therefore tend to be rather efficiently removed from the population by natural selection [6]. However, in situations where this is not the case, hybridization can lead to the collapse of closely related species into a single panmictic population [e.g. [7]], or to speciation events when some form of reproductive isolation between parental species and hybrids emerges [3-5].

In haplodiploid eusocial animals such as ants, the consequences of hybridization can be strikingly different from those in other organisms. First, a diploid queen that has mated with a heterospecific haploid male will still produce purebred sons via arrhenotokous parthenogenesis, so that hybrid males will only be produced in the F2 generation. Second, potentially negative consequences of hybrid sterility can be mitigated or even avoided when hybrid individuals mostly or exclusively become non-reproductive workers [8,9]. Hybrid workers do indeed regularly occur in a variety of ant species, whereas hybrid queens and males are normally not observed [10-12]. Hybridization in ants can therefore lead to interesting evolutionary novelties, such as genetic caste determination in interdependent lineages of *Pogonomyrmex *harvester ants, where purebred females become queens and interlineage hybrids become workers [13,14]. Another recent example is a population of *Formica *wood ants with two clearly distinct male genepools and hybrid queens [15]. This stable system is maintained by strong transmission ratio distortion with respect to the sex of the offspring. In both cases there is no gene flow between the parental populations.

In the present study we investigated possible hybridization between different species of African swarm-raiding army ants, with particular focus on Kakamega Forest, Kenya, which is one of the few sites where the two closely related species, *Dorylus (Anomma) molestus *and *Dorylus (Anomma) wilverthi*, occur in sympatry. African swarm-raiding army ants (the "driver ants") are a prominent feature in afrotropical forests, where they are prime invertebrate predators with colony sizes of over ten million individuals. They form a well-supported clade within the *Dorylus *subgenus *Anomma *[16]. Like in other Hymenoptera, females (queens and workers) are diploid, while males are haploid and are produced by arrhenotokous parthenogenesis. Army ant queens, unlike the queens of most other ants, are permanently wingless, never go on a mating flight, and do not found new colonies independently. Instead, large colonies produce a reproductive brood of several thousand winged males and just a few virgin queens. Colony fission occurs when the mother queen and a large fraction of the worker-force emigrate from the nest and leave the developing reproductive brood and the remaining workers behind. All but one of the virgin queens are eliminated in yet unknown ways. After the males have eclosed, they disperse on the wing to mate with a young queen in another colony. Virgin queens, on the other hand, never leave their natal colony and mate with typically 10 - 30 foreign males that enter the colony from outside [17]. Because colonies and their queens only disperse on foot, gene flow is highly male-biased [18,19].

An interesting consequence of this idiosyncratic reproductive system is that males have to "run the gauntlet of the workers" before they can mate, so that workers have ample opportunities to choose the mates of their queen-sister [20]. If her later breeding success is affected by some of her many matings being heterospecific, the workers are under selection to influence the outcome of this mating process because the result affects their inclusive fitness. This implies that documentation of hybridization and introgression in army ants provides direct working hypotheses about the fitness of mixed genotypes. *Dorylus *colonies are headed by a single queen (monogynous), but due to the high queen-mating frequency, most workers in a colony are half-sisters, i.e. they have the same mother queen but different fathers [17].

Previous work showed that the two species at Kakamega occupy clearly distinct ecological niches, with *D. wilverthi *being essentially restricted to intact rainforest, while *D. molestus *also occurs in savannah habitats and open agricultural landscapes [21-23]. Our preliminary morphological assessments suggested that both populations at Kakamega are aberrant when compared to allopatric populations of the same species, indicating that hybridization and introgression may have taken place. That hybridization between closely related army ants may occur is also consistent with the observation that heterospecific males are occasionally found in *Dorylus *(*Anomma*) colonies [24], and with the description of the "hybrid variety" *D. sjoestedti var. sjoestedti-wilverthi *[25].

Here we use a combination of morphological, mitochondrial and nuclear genetic data to show that hybridization and introgression have occurred historically between different species of swarm-raiding army ants at a large geographic scale in East Africa, and that ongoing hybridization can be detected in *Dorylus *workers from Kakamega. Our results allow novel inferences about the evolutionary relevance of hybridization in social insects.

## Results

### (a) Morphological Analysis

**Figure 1:**
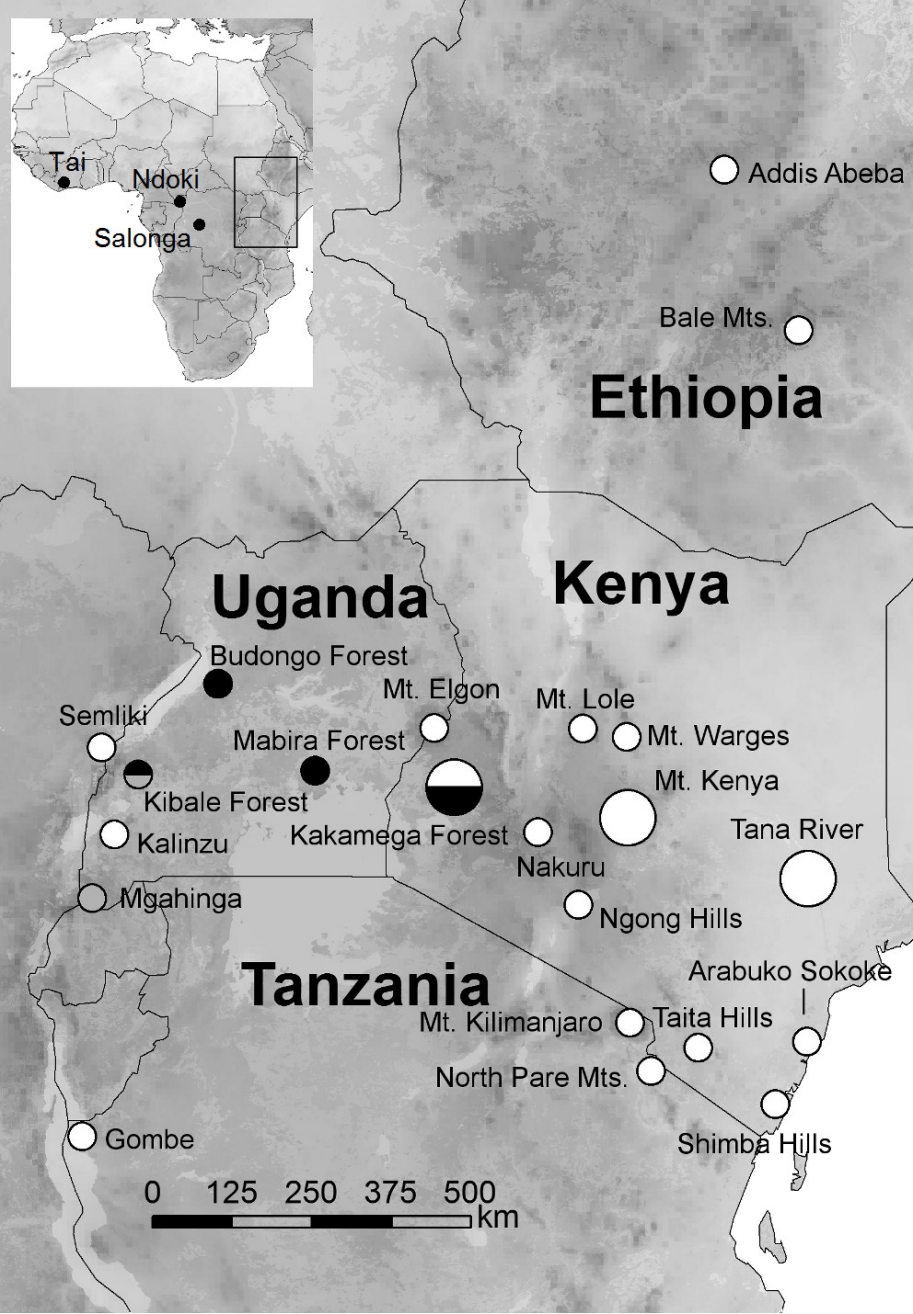
**Collection sites for *Dorylus *(*Anomma*) samples in East Africa**. White = *D. molestus*, black = *D. wilverthi*, grey = *D. terrificus*. The dots for three main study sites are plotted in larger size. The three West and Central African sites (see Table 1) where samples of *D. sjoestedti*, *D. nigricans*, and *D. emeryi *were collected are given on the inset map.

**Figure 2:**
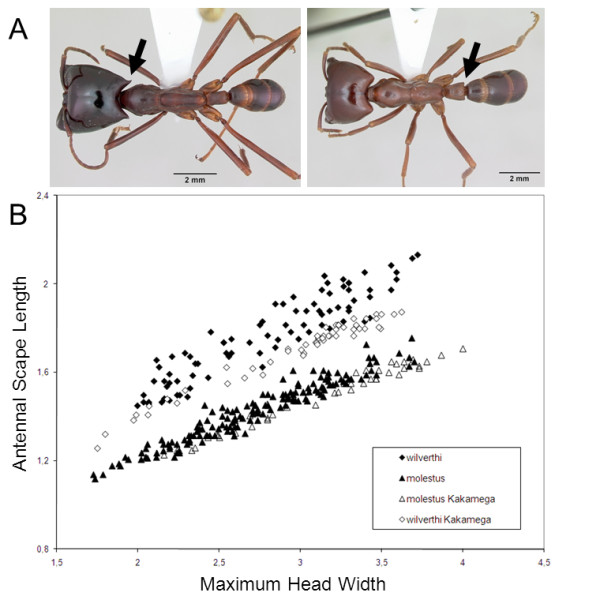
**Morphological variation among army ant populations**. (A) Photographs of *D. wilverthi *(left) and *D. molestus*. Arrows indicate diagnostic "horns" in *D. wilverthi *and petiolar tubercles in *D. molestus*. Photographs are courtesy of April Nobile and AntWeb at http://www.antweb.org. (B) The relationship between antennal scape length (SL) and maximum head width (HW) in workers of "pure" allopatric *D. molestus*, "pure" allopatric *D. wilverthi*, sympatric *D. molestus*-like individuals from Kakamega, and sympatric *D. wilverthi*-like individuals from Kakamega.

The *D. wilverthi*-like workers from Kakamega (see Figure 1 for geographic positions of study sites) had the posterior angles of the head prolonged into a raised point, but not recurved outwards as in specimens from more western populations (Figure 2a). More than half of the examined *D. molestus*-like workers lacked the characteristic petiolar tubercles. Workers of both forms at Kakamega had relatively shorter antennal scapes than the workers from the respective "pure" allopatric populations (Figure 2b). The best common regression model for the morphometric data combined was SL = 0.3039 * HW + 0.6994 (SL = antennal scape length, HW = maximum head width). Differences between the relative residuals between all four groups were highly significant (Kruskal-Wallis statistic = 280.7, p < 0.0001, Dunn's Multiple Comparison Test in all comparisons p < 0.01).

### (b) Phylogeographic Analysis

**Figure 3:**
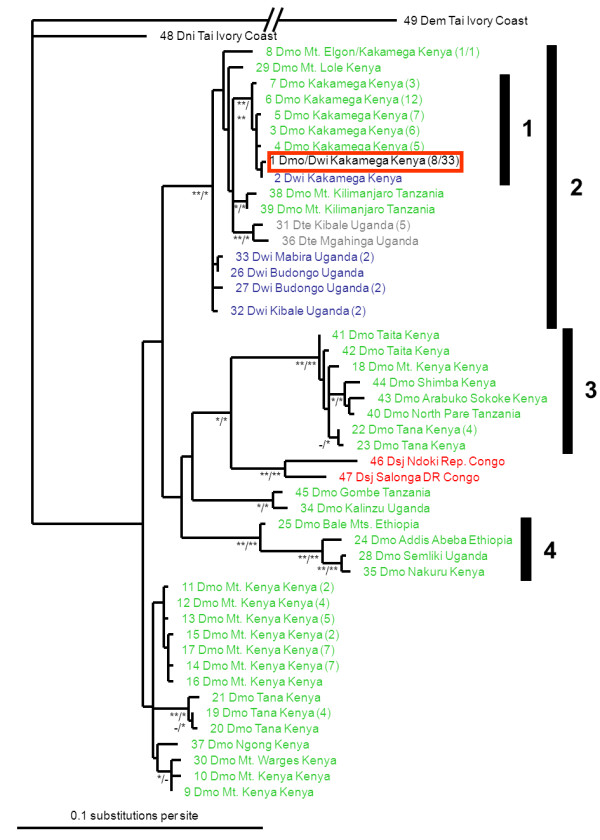
**Maximum likelihood phylogram showing relationships between mitochondrial haplotypes of East African *Dorylus *(*Anomma*) swarm-raiding army ants**. Each label gives the haplotype number (see Table 1), the abbreviated species name (Dem: *D. emeryi*; Dmo: *D. molestus *(green); Dni: *D. nigricans*; Dsj: *D. sjoestedti *(red); Dte: *D. terrificus *(grey); Dwi: *D. wilverthi *(blue)), the sample locality, and the sample size in parentheses for haplotypes that were sampled more than once. Numbered bars on the right refer to clades that are discussed in the text. Node labels represent posterior probabilities from Bayesian analyses (*: 0.95 - 0.99; **: 1.0)/maximum likelihood bootstraps (*: 0.75 - 0.9; **: > 0.9). No internal labels or a hyphen indicate support values < 0.95 and < 0.75 for the Bayesian and maximum likelihood analysis, respectively. The haplotype that is shared between the *D. molestus*-like and the *D. wilverthi*-like forms at Kakamega Forest is highlighted in a red box.

**Figure 4:**
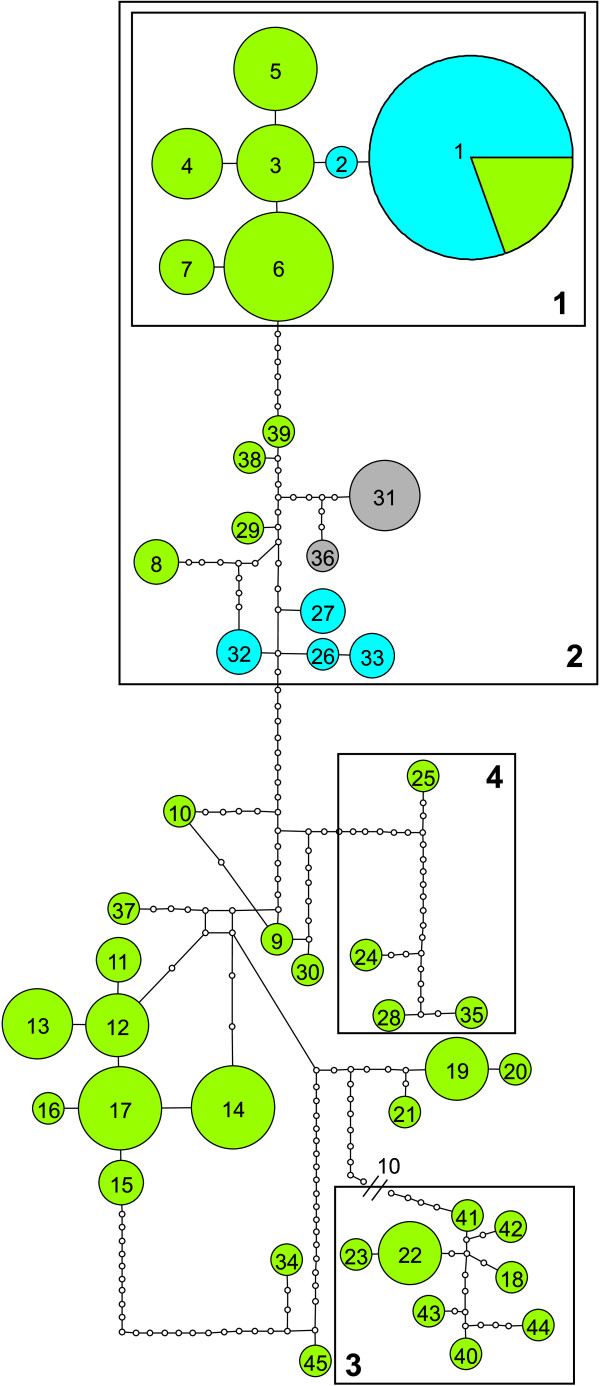
**Parsimony network of all East African haplotypes of *D. molestus*, *D. wilverthi *and *D. terrificus***. A connecting line between haplotypes represents one mutation and small open circles represent missing (inferred) haplotypes. Green haploytypes are from *D. molestus*, blue haplotypes from *D. wilverthi*, and grey haplotypes from *D. terrificus*. Size of haplotype disks is proportional to their frequencies. Boxes with numbers indicate the same haplotype clusters as shown in Figure 3.

The phylogenetic analysis of mitochondrial haplotypes resolved several well supported clades (Figures 3 and 4, Table 1). Most strikingly, neither *D. molestus *nor *D. wilverthi *were resolved as monophyletic. Instead, the mitochondrial phylogeny reflects geographical patterns rather than species affiliations: all samples from the coastal area of Kenya and Tanzania south of the Lower Tana River form a well-supported clade, extending inland to the southern slope of Mt. Kenya (clade 3 in Figures 3 and 4). The same is true for samples from Ethiopia and two of the more northern samples from Kenya and Uganda (clade 4). Samples north of and around Lake Victoria form another big clade (clade 2), which includes haplotypes from all three species that occur in the area. All common haplotypes from Kakamega form a monophyletic group (clade 1), which is nested within the larger Lake Victoria group (clade 2).

**Table 1:** List of unique mitochondrial *COII *haplotypes included in this study

**Haplotype no**.	**GenBank acc. no**.	Species	Locality	*N *colonies
1	GQ999016	*D. wilverthi*	Kakamega Forest, Kenya	33
1	GQ999023	*D. molestus*	Kakamega Forest, Kenya	8
2	GQ999019	*D. wilverthi*	Kakamega Forest, Kenya	1
3	GQ999017	*D. molestus*	Kakamega Forest, Kenya	6
4	GQ999020	*D. molestus*	Kakamega Forest, Kenya	5
5	GQ999022	*D. molestus*	Kakamega Forest, Kenya	7
6	GQ999018	*D. molestus*	Kakamega Forest, Kenya	12
7	GQ999021	*D. molestus*	Kakamega Forest, Kenya	3
8	GQ999024	*D. molestus*	Kakamega Forest, Kenya	1
8	GQ999043	*D. molestus*	Mt. Elgon, Kenya	1
9	GQ999042	*D. molestus*	Mt. Kenya (West), Kenya	1
10	EF413797	*D. molestus*	Mt. Kenya (East), Kenya	1
11	GU065701	*D. molestus*	Mt. Kenya (East), Kenya	2
12	GU065703	*D. molestus*	Mt. Kenya (East), Kenya	4
13	GU065698	*D. molestus*	Mt. Kenya (East), Kenya	5
14	GU065699	*D. molestus*	Mt. Kenya (East), Kenya	7
15	GU065702	*D. molestus*	Mt. Kenya (East), Kenya	2
16	GU065704	*D. molestus*	Mt. Kenya (East), Kenya	1
17	GU065700	*D. molestus*	Mt. Kenya (East), Kenya	7
18	GQ999037	*D. molestus*	Mt. Kenya (South), Kenya	1
19	GQ999050	*D. molestus*	Tana River (East), Kenya	4
20	GQ999052	*D. molestus*	Tana River (East), Kenya	1
21	GQ999049	*D. molestus*	Tana River (West), Kenya	1
22	GQ999032	*D. molestus*	Tana River (West), Kenya	4
23	GQ999051	*D. molestus*	Tana River (West), Kenya	1
24	GQ999027	*D. molestus*	Addis Abeba, Ethiopia	1
25	GQ999028	*D. molestus*	Bale Mts., Ethiopia	1
26	GQ999040	*D. wilverthi*	Budongo Forest, Uganda	1
27	GQ999035	*D. wilverthi*	Budongo Forest, Uganda	2
28	GQ999031	*D. molestus*	Semliki, Uganda	1
29	GQ999053	*D. molestus*	Mt. Lole, Kenya	1
30	GQ999045	*D. molestus*	Mt. Warges, Kenya	1
31	EF413804	*D. terrificus*	Kibale Forest, Uganda	5
32	EF413798	*D. wilverthi*	Kibale Forest, Uganda	2
33	GQ999034	*D. wilverthi*	Mabira Forest, Uganda	2
34	GQ999030	*D. molestus*	Kalinzu, Uganda	1
35	GQ999033	*D. molestus*	Nakuru, Kenya	1
36	GQ999039	*D. terrificus*	Mgahinga, Uganda	1
37	GQ999038	*D. molestus*	Ngong Hills, Kenya	1
38	GQ999046	*D. molestus*	Kilimanjaro, Tanzania	1
39	GQ999047	*D. molestus*	Kilimanjaro, Tanzania	1
40	GQ999048	*D. molestus*	North Pare, Tanzania	1
41	GQ999029	*D. molestus*	Taita Hills, Kenya	1
42	GQ999044	*D. molestus*	Taita Hills, Kenya	1
43	GQ999041	*D. molestus*	Arabuko Sokoke, Kenya	1
44	GQ999025	*D. molestus*	Shimba Hills, Kenya	1
45	GQ999026	*D. molestus*	Gombe, Tanzania	1
46	EF413795	*D. sjoestedti*	Ndoki, Republic of Congo	1
47	GQ999054	*D. sjoestedti*	Salonga, DR Congo	1
48	EF413803	*D. nigricans*	Tai, Ivory Coast	1
49	EF413773	*D. emeryi*	Tai, Ivory Coast	1

Haplotypes that were sampled in different species (haplotype 1) or in different populations of the same species (haplotype 8) are listed twice.

### (c) Population Structure and Divergence

We assessed the population structure and divergence among three populations of *D. molestus *(Tana River, Mt. Kenya, Kakamega Forest) and one *D. wilverthi *population (Kakamega Forest). None of the pair-wise tests for genotypic disequilibrium between microsatellite markers was significant (all p > 0.05). Pair-wise estimates of genetic differentiation between four analysed populations at nuclear microsatellites (*F_ST_*) are given in Table 2. Estimates of *F_ST _*between the *D. wilverthi*-like and the *D. molestus *populations were slightly but consistently higher than estimates between the different *D. molestus *populations (Table 2; one-tailed t-test: t = -2.33, df = 4, p = 0.04).

**Table 2:** Genetic differentiation between three populations of *D.molestus *and one sympatric population of *D. wilverthi*

	*D. molestus*			*D. wilverthi*
	**1. Mt. Kenya**	**2. Tana River**	**3. Kakamega**	**4. Kakamega**

1.	-	0.05	0.11	0.14
2.	48.9	-	0.12	0.16
3.	1.9	4.1	-	0.14
4.	3.8	1.0	18.2	-

Above diagonal: Pair-wise genetic differentiation measured as *F_ST_*. All estimates are significantly different from zero (p < 0.05) after standard Bonferroni correction for multiple comparisons. Below diagonal: The percentage of individuals that were classified as hybrids (individuals with an inferred proportion of ancestry < 0.9 in the correct source population) and "shared" between each pair of populations according to the STRUCTURE analysis (see main text for details).

The mitochondrial haplotype diversity was much lower in the *D. wilverthi*-like population (*N_a _*= 2; *R_s _*= 1.55; N = 34) than in the *D. molestus *populations (Kakamega: *N_a _*= 7; *R_s _*= 6.24; N = 42; Mt. Kenya: *N_a _*= 8; *R_s _*= 6.94; N = 29; Tana River: *N_a _*= 5; *R_s _*= 5.00; N = 11). Interestingly, the *D. wilverthi*-like population was close to fixation (allele frequency = 0.97) for a haplotype that was also common in the sympatric *D. molestus*-like population at Kakamega (allele frequency = 0.19) and was closely related to the other local *D. molestus *haplotypes (Figures 3 and 4). At the same time this is the only haplotype that is shared between any of the four populations. The second rare haplotype found in *D. wilverthi *from Kakamega is closely related to the common haplotype and differs only by a single base-pair (Figure 4).

**Figure 5:**
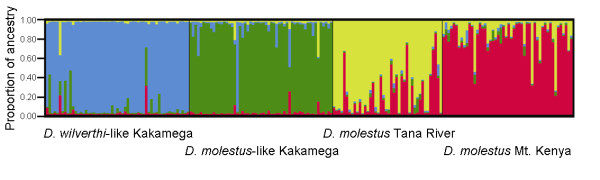
**Assignment of individuals to four populations using the program STRUCTURE without *a priori *assumptions**. Shown are assignments of individuals from the *D. wilverthi*-like population at Kakamega Forest, the *D. molestus-like *population at Kakamega Forest, and the pure *D. molestus *populations at Tana River and Mt. Kenya. The y-axis represents the proportion of each multilocus genotype that is attributable to each of the four populations. Samples are grouped along the x-axis according to their population of origin.

Replicate runs in STRUCTURE were highly consistent in every analysis. The highest likelihood scores were associated with a model with four subdivisions (*k *= 4, mean ln *L *over five replicate runs = -3185.36; 0.23 SD; for comparison: *k *= 2, -3409.86; 0.11 SD; *k *= 3, -3245.90; 0.28 SD; all *k *> 4 had lower average likelihoods than *k *= 4), although a model with five subdivisions had very similar likelihood scores (*k *= 5, -3185.56; 0.81 SD). The four clusters corresponded well to the four populations, although a significant proportion of individuals had reasonably high likelihoods of ancestry in a population other than their source population (Figure 5; Table 2). By far the strongest uncertainty over individual assignments was found between the *D. wilverthi*-like and *D. molestus*-like populations at Kakamega Forest, as well as between the Mt. Kenya and Tana River populations of *D. molestus*. When set to divide the samples in two groups (*k *= 2), STRUCTURE consistently recovered one cluster consisting of the two Kakamega populations and another cluster consisting of *D. molestus *samples from Tana River and Mt. Kenya, rather than combining the three *D. molestus *populations. At *k *= 3, STRUCTURE split the two Kakamega populations, and at *k *= 5 the Tana River + Mt. Kenya samples generally formed three clusters, while the two Kakamega clusters were retained (results not shown).

To quantify the proportion of hybrid workers at Kakamega, we arbitrarily classified individuals with an inferred proportion of ancestry < 0.9 in the correct source population as hybrids. Each hybrid individual was then determined to be "shared" between its source population and the population in which it had the highest proportion of ancestry among the non-source populations. The proportions of individuals that are "shared" between each pair of populations are given in Table 2. According to this estimate, 18.2% of the genotyped workers from Kakamega Forest were hybrids. If we lower the cut-off value for the inferred proportion of ancestry from 0.9 to 0.75, still 7.3% of the Kakamega workers were hybrids (pairwise comparisons between all populations not shown).

Results from a GENECLASS2 analysis were fully consistent with the results obtained from STRUCTURE. Five *D. wilverthi*-like individuals were assigned to the *D. molestus*-like population from Kakamega (the *D. molestus*-like population from Kakamega had the highest probability of being the source population among the four potential source populations), and one *D. molestus*-like worker from Kakamega was assigned to the *D. wilverthi*-like population. Similarly, two *D. molestus *workers from Mt. Kenya were assigned to the Tana River population, and six workers from Tana River were assigned to the Mt. Kenya population. No other cross-assignments were found.

## Discussion

### (a) Morphological analysis

The morphological and morphometric data show that two forms of swarm-raiding army ants can be distinguished at Kakamega Forest. These two forms strongly resemble *D. molestus *and *D. wilverthi *from allopatric populations, but are also significantly different from "pure" *D. molestus *and *D. wilverthi *with intermediate phenotypes for several key characters (Figure 2). This suggests that both forms have arisen from hybridization between *D. molestus *and *D. wilverthi *at Kakamega Forest. At the same time, gene-flow has not been sufficient to completely collapse the two species into a single homogeneous hybrid population.

### (b) Mitochondrial phylogeny and introgression

While mitochondrial haplotypes are clearly structured according to geographic origin (Figures 3 and 4), different *Dorylus *(*Anomma*) species are not generally recovered as monophyletic. Below we argue that this finding is best explained by the combined effects of unrecognized cryptic diversity within *D. molestus *and a complex history of mitochondrial introgression in the Lake Victoria region. *D. molestus *haplotypes tend to cluster into several well supported shallow clades, which are connected to other such clades by deeper branches (Figure 3). Furthermore, the Central African *D. sjoestedti *samples cluster with *D. molestus *samples from coastal Kenya and Tanzania, a finding that cannot be explained by introgression due to the large geographic distances and the fact that more western samples of *D. molestus *do not have haplotypes similar to those of *D. sjoestedti*. Incomplete lineage sorting seems similarly unlikely as an explanation (see below), so that this overall pattern is suggestive of unrecognized cryptic diversity within *D. molestus*.

The fact that identical or very similar haplotypes are shared between different species in spatially close populations around Lake Victoria, on the other hand, points towards a complex history of mitochondrial introgression in this narrower geographic area. The most striking example is found at Kakamega Forest, where the *D. wilverthi*-like form is close to fixation for a haplotype that is also frequent in the *D. molestus*-like form. This haplotype is nested within the clade of Kakamega *D. molestus *haplotypes (clade 1 in Figure 3). The clade of mitochondrial haplotypes from Kakamega Forest is in turn nested within a larger clade of haplotypes from the Lake Victoria region (clade 2 in Figure 3), which contains additional haplotypes from *D. molestus*, *D. terrificus*, and *D. wilverthi*. This pattern indicates a complex history of repeated mitochondrial introgression between different pairs of species.

The combination of two main factors makes it difficult to determine the original source species of the Lake Victoria haplotypes: 1. We presently lack haplotype information for populations of *D. wilverthi *and *D. terrificus *that have clearly not undergone mitochondrial introgression, i.e. we are missing "true" *D. wilverthi *and *D. terrificus *haplotypes in our analysis. 2. Mitochondrial DNA undergoes frequent selective sweeps, and recurrent sweeps are likely to mask previous introgression events [26,27].

During Pleistocene and Holocene glacial cycles [28,29], the Lake Victoria region has seen recurrent expansions and retractions of forest and savannah habitat [30-32], so it is safe to assume that the distributional ranges of rainforest specialists like *D. wilverthi *and savannah/forest generalists like *D. molestus *have changed accordingly. The two species probably broadly overlapped during glacial periods of maximum forest expansion, when Kakamega Forest was connected to Ugandan rainforests [30], while *D. wilverthi *has been restricted to forest islands during interglacial periods as is the case at present. Given this complex recent biogeographic history, it will also be challenging to associate the observed cases of introgressive hybridization with discrete historic events.

It is interesting to note that only a single mitochondrial haplotype is shared between the two forms at Kakamega, while essentially all other haplotypes are restricted to the *D. molestus*-like form. One possible explanation is that mitochondrial gene-flow at Kakamega goes back to a single or very few events, which by chance involved only that particular haplotype. Alternatively, the pattern could be explained by selection. For example, all other *D. molestus*-like haplotypes, or potential cytoplasmic factors coupled to those haplotypes (like *Wolbachia *infections [33]), might have epistatic incompatibilities with parts of the *D. wilverthi *nuclear genome. That the reduced mitochondrial diversity in the *D. wilverthi*-like form at Kakamega is simply due to a recent genetic bottleneck seems unlikely because diversity at nuclear microsatellite loci is not reduced compared to other populations (Table 3).

**Table 3:** Number of alleles (*N_a_*), expected heterozygosity (*H_s_*), allelic richness (*R_s_*), and inbreeding coefficient (*F_IS_*) estimated for five microsatellite loci and three populations of *D.molestus *and one sympatric population of *D. wilverthi*

	*D. molestus*												*D. wilverthi*			
	
	Mt. Kenya (N = 50)				Tana River (N = 42)				Kakamega (N = 55)				Kakamega (N = 55)			
**Locus**	***N***_***a***_	***H***_***s***_	***R***_***s***_	***F***_***IS***_	***N***_***a***_	***H***_***s***_	***R***_***s***_	***F***_***IS***_	***N***_***a***_	***H***_***s***_	***R***_***s***_	***F***_***IS***_	***N***_***a***_	***H***_***s***_	***R***_***s***_	***F***_***IS***_

DmoB	4	0.56	3.82	-0.18	3	0.55	3.00	0.09	5	0.65	4.97	0.05	7	0.55	6.84	0.04
DmoC	4	0.57	4.00	0.07	5	0.68	5.00	0.28	5	0.69	5.00	0.11	4	0.52	3.75	0.23
DmoD	5	0.77	5.00	-0.05	8	0.85	8.00	-0.04	8	0.81	7.49	0.02	7	0.74	6.67	0.14
DmoG	11	0.86	10.46	0.19*	12	0.86	11.98	0.11	9	0.82	8.49	-0.02	6	0.72	6.00	-0.04
DmoO	9	0.86	8.97	0.26*	8	0.71	7.95	0.07	9	0.77	8.99	-0.04	9	0.79	8.67	0.10

mean	6.6	0.72	6.45	0.06	7.2	0.73	7.19	0.10	7.2	0.75	6.99	0.02	6.6	0.66	6.39	0.09

*F_IS _*estimates significantly different from zero (p < 0.05 after standard Bonferroni correction for multiple comparisons) are marked with an asterisk.

Another noteworthy case of apparent mitochondrial introgression was found in the population from Tana River, where haplotypes from both sides of the river tend to cluster in clearly distinct clades (Figures 3 and 4) although, according to microsatellite data (see below), all samples belong to a single population. This suggests that at least one side of the river has captured haplotypes from a more distantly related *D. molestus *clade. This strong genetic structure at the maternally inherited mitochondrial locus, and a lack thereof at biparentally inherited nuclear loci, stems from the fact that the permanently wingless army ant queens cannot cross water barriers, while the winged males readily do so [18].

Alternative explanations for a lack of monophyly between closely related species usually involve the retention of ancestral polymorphisms or incomplete lineage sorting. However, these mechanisms are less likely to apply to mtDNA as compared to nuclear DNA because ancestral polymorphisms will be lost more quickly due to genetic drift given the smaller effective population size of mtDNA and because mtDNA in insects evolves significantly faster than nuclear DNA (e.g. [34]). On the other hand, mtDNA seems to be particularly prone to introgression, at least in species where males are the heterogametic or haploid sex (e.g. [34-37]). One reason for this is that in such species males tend to suffer more from hybridization than females (Haldane's rule; [38]), so that gene-flow will be more restricted at nuclear loci relative to maternally inherited mitochondrial loci. Furthermore, if incomplete lineage sorting occurs at random it should not produce an obvious correlation with geographic distribution (e.g. [39]), as we observed in East African *Dorylus *(*Anomma*) species. Under incomplete lineage sorting, the minimum divergence time between sequences is given by the time elapsed since speciation [40]. In the absence of gene flow, mutations should therefore accumulate quickly in *cytochrome oxidase II *haplotypes, which have high mutation rates of ca. 1.5% per million years in insects (e.g. [41]). Given the strong geographic signal in our mtDNA phylogeny (Figures 3 and 4), the general lack of overlapping haplotypes between neighbouring conspecific populations, and the fact that sympatric species at Kakamega share identical haplotypes for fast evolving mitochondrial genes, we conclude that introgressive hybridization between army ant species is clearly the best explanation for the observed haplotype distribution in the Lake Victoria area.

### (c) Differentiation and hybridization at nuclear DNA

The two morphological forms at Kakamega Forest can be clearly distinguished based on the nuclear microsatellite loci. This again shows that species boundaries have not become completely blurred, which would be expected if gene flow had been significant over extended periods of time. On the other hand, despite genetic differentiation among species being consistently higher than between conspecific populations (Table 2), a large proportion of workers at Kakamega could not be unequivocally assigned to one of the two forms by either STRUCTURE or GENECLASS2 (Figure 5). At the same time, pair-wise assignments between populations were far less problematic, even in conspecific comparisons. The only exception was the Tana River/Mt. Kenya comparison, which showed very weak differentiation making assignments difficult. The reason for this genetic similarity may well be that these two populations were connected via contiguous gallery forests along the Tana River until the recent past. Overall, this implies that a considerable proportion of workers at Kakamega Forest are hybrids between *D. molestus *and *D. wilverthi*.

## Conclusions

This study provides conclusive evidence that introgressive hybridization has occurred between different *Dorylus (Anomma) *species. This is one of the few cases of documented gene flow between species of eusocial Hymentoptera ([11]; but see [42,43] for other recent examples) and shows that reproductive isolation between species of East African swarm-raiding army ants is incomplete. The frequent occurrence of hybrid workers at Kakamega means that the two local forms still mate regularly today. However, it is important to keep in mind that hybrid workers do not necessarily translate into gene flow, because social insect workers normally do not reproduce. This also applies to *Dorylus *(*Anomma*) [19,44]. The general notion that hybrid workers are common between several pairs of closely related ant species, while hybrid queens and males are extremely uncommon [11], might explain why the two forms at Kakamega have retained their distinct morphology and ecological niche space, instead of having collapsed into a single homogeneous hybrid population. Hybridization in army ants is particularly surprising because, unlike in most social insects where mating occurs outside the nest, workers have control over which males obtain access to a virgin queen. Therefore, if hybridization would imply reduced colony fitness, workers should be under strong selection to recognize heterospecific males and prevent them from mating. This suggests that hybridization in army ants entails little or no fitness costs or might even be adaptive under certain circumstances. Future work will have to investigate the stability of the hybrid zone at Kakamega over time, and the effects of hybridization on the actual genotypes of *Dorylus *queens and males, to clarify the precise pattern of hybridization and the amount of ongoing gene-flow between the two sympatric forms.

## Materials and methods

### (a) Sampling and morphological analysis

To study patterns of mitochondrial differentiation between species and populations of East African *Dorylus *(*Anomma*) army ants, we collected 151 colony samples (one individual per colony) for mitochondrial DNA sequencing from 24 localities, representing four morphologically distinguishable species, *D. molestus*, *Dorylus sjoestedti*, *Dorylus terrificus*, and *D. wilverthi *(Figure 1; Table 1). A single West African sample of *Dorylus nigricans *was also included. A haplotype of the West African species *Dorylus emeryi *served as outgroup, as this species belongs to the sister clade of the swarm-hunting *Anomma *"driver ants" [16].

Samples for microsatellite genotyping (one worker per colony) were collected from four populations at three different sites in Kenya: Kakamega Forest (where *D. molestus*-like and *D. wilverthi*-like forms are sympatric; N = 55 each), the eastern slope of Mt. Kenya (*D. molestus*; N = 50), and Lower Tana River (*D. molestus*; N = 42). Subsets of these samples were also sequenced for mitochondrial DNA (see Table 1). The Mt. Kenya population is geographically situated halfway in between the two other populations (Figure 1). All samples were collected and stored in ethanol.

We identified samples morphologically using the diagnostic criteria given in the original species descriptions and in [45]. Furthermore, samples were directly compared with type specimens. *D. molestus*, *D. terrificus*, and *D. sjoestedti*, which currently have the status of subspecies of *D. nigricans*, were treated as species according to [45,46], and the results of an ongoing taxonomic revision of the *Dorylus *(*Anomma*) swarm-raiding army ants (C. Schöning *et al. *unpublished). Importantly, *D. wilverthi *and *D. molestus *workers are easily distinguished: *D. wilverthi *workers have the posterior angles of the head uniquely prolonged into a raised, slightly outwardly recurved point ("horns"; Figure 2a), while *D. molestus *workers have posteroventral extensions ("tubercles") on the petiole (Figure 2a) and significantly shorter appendages than *D. wilverthi *[47,48]. To quantify morphological variation we measured antennal scape length (SL) in relation to maximum head width (HW) for large workers (HW > 1.5 mm) in four groups: *D. molestus*-like individuals from Kakamega (N = 54 workers from 10 colonies), *D. wilverthi*-like individuals from Kakamega (N = 45 workers from nine colonies), "pure" allopatric *D. molestus *(N = 157 workers from 31 colonies belonging to 20 populations across the species' range), and "pure" allopatric *D. wilverthi *(N = 85 workers from 16 colonies belonging to 10 populations across the species' range). Measurements were taken with a MS5 Leica stereomicroscope fitted with an ocular micrometer. Data were analysed following Schöning *et al. *[47].

### (b) DNA extraction, amplification, sequencing, and microsatellite genotyping

DNA for sequencing was extracted from 1-2 worker legs using standard QIAGEN^® ^(DNeasy^®^) and MACHEREY-NAGEL (Nucleo Spin^®^Tissue) kits. A mitochondrial fragment of the *cytochrome oxidase II *(*COII*) gene was amplified and sequenced using primers tRNALeu [16] and Barbara [49] as has been described earlier [16]. Sequencing reactions were either performed in house or purified PCR products were sent to a commercial sequencing facility (Macrogen, Korea). The final alignment used in this study consisted of 548 bp of *COII *sequence. GenBank accession numbers and details for sequenced samples are given in Table 1.

DNA for microsatellite genotyping was extracted by boiling 1-2 worker legs in 100 μl of 5% Chelex 100 (Bio-Rad). A total of 202 individuals were genotyped at five microsatellite loci (DmoB, DmoC, DmoD, DmoG, DmoO) as has been described previously [50].

### (c) Phylogeographic Analyses

Phylogenetic analyses of mitochondrial sequences were conducted in a Bayesian framework using the program MrBayes 3.1.2 [51,52], and in a maximum likelihood framework using the program GARLI 0.96 [53]. We implemented a general time reversible model with gamma-distributed rate heterogeneity (GTR+G), which was selected as the most appropriate model for our data by both hierarchical Likelihood Ratio Tests and the Akaike Information Criterion in MrModeltest 2.3 [54].

To assure convergence of Markov Chain Monte Carlo runs in MrBayes we repeated the analysis from two independent starting trees. One cold and three heated chains were run in parallel for 20*10^6 ^generations and trees were sampled every 100 generations. Average standard deviations of split frequencies were consistently < 0.01 after 13*10^6 ^generations, indicating that the independent runs had converged. Accordingly, the first 130,000 trees from each run were discarded as burn-in. Based on this sample of 140,002 trees from both runs combined, all potential scale reduction factors for model parameters were < 1.02, indicating effective sampling from the posterior probability distribution. A consensus phylogram with posterior probabilities based on both runs was calculated in MrBayes.

We initially performed three independent runs in GARLI setting the number of generations after which the run is terminated if no new significantly better scoring topology has been found to 30,000, and otherwise using default parameter settings. We then performed a run with 1,000 bootstrap replicates setting the above parameter at 10,000.

A mitochondrial haplotype network for all East African haplotypes of *D. molestus*, *D. wilverthi *and *D. terrificus *was constructed using a statistical parsimony approach [55] implemented in TCS version 1.21 [56]. The connection limit in the TCS program was set at unlimited.

### (d) Population Structure and Divergence

The number of alleles per microsatellite locus (*N_a_*), expected heterozygosity (*H_s_*), allelic richness corrected for sample size (*R_s_*), and the inbreeding coefficient *F_IS _*were estimated for each population using the program FSTAT 2.9.3.2 [57]. *F_IS _*was tested for significant deviation from zero using 400 randomisations to assess Hardy Weinberg equilibrium. The same program was used to test for genotypic disequilibrium between all pairs of microsatellite loci within each population (Table 3). Furthermore, we used FSTAT to calculate pair-wise *F_ST _*as an estimate of genetic differentiation at microsatellite loci between populations and species. FSTAT was also used to calculate *N_a _*and *R_s _*for mitochondrial haplotypes.

We used two different model-based Bayesian methods to study hybridization between *D. molestus *and *D. wilverthi *at Kakamega. First, the clustering method of the program STRUCTURE 2.2 [58] was used to infer population structure at microsatellite loci without prior information on species assignments based on sample locality and morphology. The data from all four populations were combined in this analysis. To assure convergence and consistency of results, we conducted five replicate runs with different random seeds for each *k*, the number of subdivisions, ranging from one to 10, under an admixture model with correlated allele frequencies [59]. Markov chains were run for 10^6 ^generations and the first 10^5 ^generations were discarded as burn-in. Estimates of hybrid frequencies were based on individual probabilities of having ancestry in populations other than the source population. The comparisons between conspecific populations were used as a control to assure that the analyses had sufficient power in assigning individuals to different clusters given the observed levels of genetic differentiation between populations. More recent and potentially ongoing gene-flow between conspecific populations should generally result in more ambiguous assignments of individuals to populations as compared to genetically isolated heterospecific populations, irrespective of whether these occur in sympatry or not. We used the program DISTRUCT 1.1 [60] to graphically display STRUCTURE results (Figure 4).

Second, we used the assignment methods in the program GENECLASS2 [61] to estimate the likelihood of origin for each individual in each potential source population. All four populations were analyzed in a single dataset. To calculate likelihoods, we chose a Bayesian criterion with a Dirichlet prior distribution for allele frequencies [62] using the resampling method of Paetkau *et al. *[63]. The number of simulated individuals for probability computation was set at 10,000.

## Competing interests

The authors declare that they have no competing interests.

## Authors' contributions

DJCK, MKP, CS, JJB designed the study an acquired funding. MKP and CS collected all army ant samples and identified them. DJCK and MKP conducted the molecular genetic studies and analysed the data. CS conducted the morphometric analyses. DJCK wrote the first version of the paper. All authors contributed to and approved the final manuscript.

## Authors' information

DJCK is the head of the Laboratory of Insect Social Evolution at The Rockefeller University. He is broadly interested in the evolution of social insects, and army ants are his main study systems. MKP is a postdoctoral ecologist at the Department of Animal Ecology and Tropical Biology, University of Würzburg, Germany. He studies the ecology and conservation of tropical rainforest animals. CS is a postdoctoral fellow at the Länderinstitut für Bienenkunde in Hohen Neuendorf, Germany, where he presently works on honeybees. He has a long-standing interest in the ecology and evolution of African army ants. JJB is the director of the Centre for Social Evolution at the University of Copenhagen.

## References

[B1] Rieseberg LH Hybrid origins of plant species Annu Rev Ecol Syst 1997 28 359 389 10.1146/annurev.ecolsys.28.1.359

[B2] Dowling TE Secor CL The role of hybridization and introgression in the diversification of animals Annu Rev Eco Syst 1997 28 593 619 10.1146/annurev.ecolsys.28.1.593

[B3] Seehausen O Hybridization and adaptive radiation Trends Ecol Evol 2004 19 198 207 10.1016/j.tree.2004.01.003 16701254

[B4] Mallet J Hybrid speciation Nature 2007 446 279 283 10.1038/nature05706 17361174

[B5] Mavárez J Linares M Homoploid hybrid speciation in animals Mol Ecol 2008 17 4181 4185 10.1111/j.1365-294X.2008.03898.x 19378399

[B6] Burke JM Arnold ML Genetics and the fitness of hybrids Annu Rev Genet 2001 35 31 52 10.1146/annurev.genet.35.102401.085719 11700276

[B7] Taylor EB Boughman JW Groenenboom M Sniatynski M Schluter D Gow JL Speciation in reverse: morphological and genetic evidence of the collapse of a three-spined stickleback (*Gasterosteus aculeatus*) species pair Mol Ecol 2006 15 343 355 16448405 10.1111/j.1365-294X.2005.02794.x

[B8] Anderson KE Linksvayer TA Smith CR The causes and consequences of genetic caste determination in ants (Hymenoptera: Formicidae) Myrmecol News 2008 11 119 132

[B9] Schwander T Lo N Beekman M Oldroyd BP Keller L Nature versus nurture in social insect caste differentiation Trends Ecol Evol 2010 25 275 282 10.1016/j.tree.2009.12.001 20106547

[B10] Umphrey GJ Sperm parasitism in ants: selection for interspecific mating and hybridization Ecology 2006 87 2148 2159 10.1890/0012-9658(2006)87[2148:SPIASF]2.0.CO;2 16995614

[B11] Feldhaar H Foitzik S Heinze J Lifelong commitment to the wrong partner: hybridization in ants Philos T R Soc B 2008 363 2891 2899 10.1098/rstb.2008.0022 PMC2606732 18508757

[B12] Van der Have TM Pedersen JS Boomsma JJ Mating, hybridisation and introgression in *Lasius *ants (Hymenoptera, Formicidae) Myrmecological News 2010 15 109 115

[B13] Volny VP Gordon DM Genetic basis for queen-worker dimorphism in a social insect Proc Natl Acad Sci USA 2002 99 6108 6111 10.1073/pnas.092066699 11972049 PMC122910

[B14] Helms Cahan SH Keller L Complex hybrid origin of genetic caste determination in harvester ants Nature 2003 424 306 309 10.1038/nature01744 12867980

[B15] Kulmuni J Seifert B Pamilo P Segregation distortion causes large-scale differences between male and female genomes in hybrid ants Proc Natl Acad Sci USA 2010 107 7371 7376 10.1073/pnas.0912409107 20368452 PMC2867678

[B16] Kronauer DJC Schöning C Vilhelmsen L Boomsma JJ A molecular phylogeny of *Dorylus *army ants provides evidence for multiple evolutionary transitions in foraging niche BMC Evol Biol 2007 7 56 10.1186/1471-2148-7-56 17408491 PMC1852301

[B17] Kronauer DJC Schöning C Pedersen JS Boomsma JJ Gadau J Extreme queen-mating frequency and colony fission in African army ants Mol Ecol 2004 13 2381 2388 10.1111/j.1365-294X.2004.02262.x 15245410

[B18] Berghoff SM Kronauer DJC Edwards KJ Franks NR Dispersal and population structure of a New World predator, the army ant *Eciton burchellii* J Evolution Biol 2008 21 1125 1132 10.1111/j.1420-9101.2008.01531.x 18422531

[B19] Kronauer DJC Schöning C d'Ettorre P Boomsma JJ Colony fusion and worker reproduction after queen loss in army ants Proc Roy Soc B 2010 277 755 763 10.1098/rspb.2009.1591 PMC2842746 19889701

[B20] Franks NR Hölldobler B Sexual competition during colony reproduction in army ants Biol J Linn Soc 1987 30 229 243 10.1111/j.1095-8312.1987.tb00298.x

[B21] Schöning C Kinuthia W Boomsma JJ Does the afrotropical army ant *Dorylus (Anomma) molestus *go extinct in fragmented forests? J East Afr Nat Hist 2006 95 163 179 10.2982/0012-8317(2006)95[163:DTAAAD]2.0.CO;2

[B22] Peters MK Okalo B Severe declines of ant-following birds in African rainforest fragments are facilitated by a subtle change in army ant communities Biol Conserv 142 2050 2058

[B23] Peters MK Fischer G Schaab G Kraemer M Species compensation maintains abundance and raid rates of African swarm-raiding army ants in rainforest fragments Biol Conserv 2009 142 668 675 10.1016/j.biocon.2008.11.021

[B24] Raignier A Van Boven JKA Ceusters R Schmidt GH Der Polymorphismus der afrikanischen Wanderameisen unter biometrischen und biologischen Gesichtspunkten Sozialpolymorphismus bei Insekten. Probleme der Kastenbildung im Tierreich 1974 Stuttgart: Wissenschaftliche Verlagsgesellschaft 668 693

[B25] Wasmann E Neue Anpassungstypen bei Dorylinengästen Afrikas (Col. Staphylinidae). (218. Beitrag zur Kenntnis der Myrmekophilen.) Zeitschr Wissenschaftl Zool 1917 17 257 360

[B26] Ballard JWO Whitlock MC The incomplete natural history of mitochondria Mol Ecol 2004 13 729 744 10.1046/j.1365-294X.2003.02063.x 15012752

[B27] Bazin E Glemin S Galtier N Population size does not influence mitochondrial genetic diversity in animals Science 2006 312 570 572 10.1126/science.1122033 16645093

[B28] Bintanja R Van de Wal RSW Oerlemans J Modelled atmospheric temperatures and global sea levels over the past million years Nature 2005 437 125 128 10.1038/nature03975 16136140

[B29] Caputo R Sea-level curves: perplexities of an end-user in morphotectonic applications Global Planet Change 2007 57 417 423 10.1016/j.gloplacha.2007.03.003

[B30] Wagner P Köhler J Schmitz A Böhme W The biogeographical assignment of a west Kenyan rain forest remnant: further evidence from analysis of its reptile fauna J Biogeogr 2008 35 1349 1361 10.1111/j.1365-2699.2008.01883.x PMC7167171 32336867

[B31] Moreau RE Pleistocene climatic changes and the distribution of life in East Africa J Ecol 1933 21 415 435 10.2307/2256590

[B32] Mahaney WC Quaternary and environmental research on East African mountains 1989 Rotterdam: AA. Balkema

[B33] Hilgenboecker K Hammerstein P Schlattmann P Telschow A Werren JH How many species are infected with *Wolbachia*? - a statistical analysis of current data FEMS Microbiol Let 2008 281 215 220 10.1111/j.1574-6968.2008.01110.x PMC2327208 18312577

[B34] Bachtrog D Thornton K Clark A Andolfatto P Extensive introgression of mitochondrial DNA relative to nuclear genes in the *Drosophila yakuba *species group Evolution 2006 60 292 302 16610321

[B35] Powell J Interspecific cytoplasmic gene flow in the absence of nuclear gene flow: evidence from *Drosophila* Proc Natl Acad Sci USA 1983 80 492 495 10.1073/pnas.80.2.492 6300849 PMC393404

[B36] Roca A Georgiadis N O'Brien S Cytonuclear genomic dissociation in African elephant species Nat Genet 2005 37 96 100 15592471 10.1038/ng1485

[B37] Linnen CR Farrell BD Mitonuclear discordance is caused by rampant mitochondrial introgression in *Neodiprion *(Hymenoptera: Diprionidae) sawflies Evolution 2007 61 1417 1438 10.1111/j.1558-5646.2007.00114.x 17542850

[B38] Koevoets T Beukeboom LW Genetics of postzygotic isolation and Haldane's rule in haplodiploids Heredity 2009 102 16 23 10.1038/hdy.2008.44 18523445

[B39] Berthier P Excoffier L Ruedi M Recurrent replacement of mtDNA and cryptic hybridization between two sibling bat species *Myotis myotis *and *Myotis blythii* Proc Roy Soc B 2006 273 3101 3109 10.1098/rspb.2006.3680 PMC1679893 17018432

[B40] Joly S McLenachan PA Lockhart PJ A statistical approach for distinguishing hybridization and incomplete lineage sorting Am Nat 2009 174 E54 E70 10.1086/600082 19519219

[B41] Schoville SD Roderick GK Evolutionary diversification of cryophilic *Grylloblatta *species (Grylloblattodea: Grylloblattidae) in alpine habitats of California BMC Evol Biol 2010 10 163 10.1186/1471-2148-10-163 20525203 PMC2898686

[B42] Ross KG Gotzek D Ascunce MS Shoemaker DD Species delimitation: a case study in a problematic ant taxon Syst Biol 2010 59 162 184 10.1093/sysbio/syp089 20525628

[B43] Seifert B Kulmuni J Pamilo P Independent hybrid populations of *Formica polyctena *X *rufa *wood ants (Hymenoptera: Formicidae) abound under conditions of forest fragmentation Evol Ecol 2010 24 1219 1237 10.1007/s10682-010-9371-8

[B44] Kronauer DJC Schöning C Boomsma JJ Male parentage in army ants Mol Ecol 2006 15 1147 1151 10.1111/j.1365-294X.2005.02850.x 16599973

[B45] Gotwald WH Schaefer RF Taxonomic implications of doryline worker ant morphology: Dorylus subgenus *Anomma *(Hymenoptera: Formicidae) Sociobiology 1982 7 187 204

[B46] Gotwald WH Predatory behavior and food preferences of driver ants in selected African habitats Ann Entomol Soc Am 1974 67 877 886

[B47] Schöning C Kinuthia W Franks NR Evolution of allometries in the worker caste of *Dorylus *army ants Oikos 2005 110 231 240 10.1111/j.0030-1299.2005.13672.x

[B48] Schöning C Humle T Möbius Y McGrew WC The nature of culture: technological variation in chimpanzee predation on army ants revisited J Hum Evol 2008 55 48 59 10.1016/j.jhevol.2007.12.002 18275983

[B49] Simon C Frati F Beckenbach A Crespi B Liu H Flook P Evolution, weighting, and phylogenetic utility of mitochondrial gene sequences and a compilation of conserved polymerase chain reaction primers Ann Entomol Soc Am 1994 87 651 701

[B50] Kronauer DJC Boomsma JJ Gadau J Microsatellite markers for the driver ant *Dorylus (Anomma) molestus* Mol Ecol Notes 2004 4 289 290 10.1111/j.1471-8286.2004.00645.x

[B51] Huelsenbeck JP Ronquist F MRBAYES: Bayesian inference of phylogeny Bioinformatics 2001 17 754 755 10.1093/bioinformatics/17.8.754 11524383

[B52] Ronquist F Huelsenbeck JP MRBAYES 3: Bayesian phylogenetic inference under mixed models Bioinformatics 2003 19 1572 1574 10.1093/bioinformatics/btg180 12912839

[B53] Zwickl DJ Genetic algorithm approaches for the phylogenetic analysis of large biological sequence datasets under the maximum likelihood criterion PhD thesis 2006 The University of Texas at Austin

[B54] Nylander JAA MrModeltest v2 Program distributed by the author 2004 Evolutionary Biology Centre, Uppsala University

[B55] Templeton AR Crandall KA Sing CF A cladistics analysis of phenotypic associations with haplotypes inferred from restriction endonuclease mapping and DNA sequence data III. Cladogram estimation Genetics 1992 132 619 633 1385266 10.1093/genetics/132.2.619 PMC1205162

[B56] Clement M Posada D Crandall KA TCS: a computer program to estimate gene genealogies Molecular Ecology 2000 9, 1657 1659 11050560 10.1046/j.1365-294x.2000.01020.x

[B57] Goudet J FSTAT, a program to estimate and test gene diversities and fixation indices (version 2.9.3.2) http://www2.unil.ch/popgen/softwares/fstat.htm

[B58] Pritchard JK Stephens M Donnelly P Inference of population structure using multilocus genotype data Genetics 2000 155 945 959 10835412 10.1093/genetics/155.2.945 PMC1461096

[B59] Falush D Stephens M Pritchard JK Inference of population structure: Extensions to linked loci and correlated allele frequencies Genetics 164 1567 1587 10.1093/genetics/164.4.1567 PMC1462648 12930761

[B60] Rosenberg NA DISTRUCT: a program for the graphical display of population structure Mol Ecol Notes 2004 4 137 138

[B61] Piry S Alapetite A Cornuet JM Paetkau D Baudouin L Estoup A GENECLASS2: A software for genetic assignment and first-generation migrant detection J Hered 2004 95 536 539 10.1093/jhered/esh074 15475402

[B62] Rannala B Mountain JL Detecting immigration by using multilocus genotypes Proc Natl Acad Sci USA 1997 94 9197 9201 10.1073/pnas.94.17.9197 9256459 PMC23111

[B63] Paetkau D Slade R Burden M Estoup A Genetic assignment methods for the direct, real-time estimation of migration rate: a simulation-based exploration of accuracy and power Mol Ecol 2004 13 55 65 10.1046/j.1365-294X.2004.02008.x 14653788

